# The hidden cost of chronic fatigue to patients and their families

**DOI:** 10.1186/1472-6963-10-56

**Published:** 2010-03-04

**Authors:** Ramon Sabes-Figuera, Paul McCrone, Mike Hurley, Michael King, Ana Nora Donaldson, Leone Ridsdale

**Affiliations:** 1 Centre for the Economics of Mental Health, Health Services Research Department, Institute of Psychiatry, King's College, London, UK; 2 Academic Department of Physiotherapy Health and Social Care Research Division King's College London, London, UK; 3 Department of Mental Health Sciences, Hampstead Campus, University College London, London, UK; 4 King's College London Dental Institute, King's College, London, UK; 5 Unit of Neurology and General Practice, Department of Clinical Neuroscience, Institute of Psychiatry, King's College London, London, UK

## Abstract

**Background:**

Nearly 1 in 10 in the population experience fatigue of more than six months at any one time. Chronic fatigue is a common reason for consulting a general practitioner, and some patients report their symptoms are not taken seriously enough. A gap in perceptions may occur because doctors underestimate the impact of fatigue on patients' lives. The main aim of the study is to explore the economic impact of chronic fatigue in patients seeking help from general practitioners and to identify characteristics that explain variations in costs.

**Methods:**

The design of study was a survey of patients presenting to general practitioners with unexplained chronic fatigue. The setting were 29 general practice surgeries located in the London and South Thames regions of the English National Health Service. Use of services over a six month period was measured and lost employment recorded. Regression models were used to identify factors that explained variations in these costs.

**Results:**

The mean total cost of services and lost employment across the sample of 222 patients was £3878 for the six-month period. Formal services accounted for 13% of this figure, while lost employment accounted for 61% and informal care for 26%. The variation in the total costs was significantly related to factors linked to the severity of the condition and social functioning.

**Conclusions:**

The economic costs generated by chronic fatigue are high and mostly borne by patients and their families. Enquiry about the functional consequences of fatigue on the social and occupational lives of patients may help doctors understand the impact of fatigue, and make patients feel better understood.

## Background

A quarter of the population report tiredness as being a recent health problem [1], and for about 1 in 10 people such fatigue is chronic lasting more than six months [2]. If fatigue lasts longer than 6 months, causes important disability, is accompanied by other symptoms, but is unexplained it has been labelled chronic fatigue syndrome/myalgic encephalitis (CFS/ME) [3]. Fatigue is seen by many patients as a consequence of modern life and is usually self-managed. A diary study of women patients showed that only 1 in 400 episodes led to a consultation with a doctor [4]. When they do seek clinical help, most people believe there is a physical rather than a psychological cause to their fatigue, although a physical cause is only identified by GPs in about a fifth of cases [5]. Physical as well as psychosocial factors need to be explored before symptoms can be defined as 'medically unexplained' [6]. Doctors may take physical and psychological factors into account. However patients report that doctors do not take their experience of fatigue symptoms and its impact seriously [3,7,8]. One of the reasons for this could be that doctors do not see and therefore are not aware of the real impact of the condition in the life of the patients. The perception of a gap in understanding can undermine the therapeutic relationship [3,7,8]. Some of the gap in perceptions may be caused by lack of enquiry or awareness of the social and economic cost.

Fatigue has been shown to result in high economic costs to society, especially because of its impact on employment and the need for families and friends to spend time caring for the individual [9]. The aims of this paper are (i) to explore the costs of chronic fatigue in a sample of patients attending general practitioners in the south east of England and (ii) to identify the factors associated with these costs. Such cost estimates allow the 'burden' of the condition to be estimated and can serve as a comparator for costs that arise after treatments have been delivered.

## Methods

### Setting and sample

Participants for the study were recruited from 29 general practice surgeries in the London and South Thames National Health Service region. The practices covered a population of approximately 236,000 people. Between November 2003 and October 2007 patients presenting at these general practice surgeries complaining of chronic fatigue were referred by doctors if they fulfilled the following criteria. Patients had to: (i) have had fatigue for more than 3 months, (ii) have a score greater than 4 on a 11-item self-reported Chalder fatigue scale which has been shown to be valid and reliable [10], (iii) be aged between 16 and 75, and (iv) have had no recent change to any drug regimen, normal full blood count, erythrocyte sedimentation rate and thyroid function test. The duration criteria of more than 3 months (instead of the usual 6 months used by specialists as a minimum criteria in the diagnosis of chronic fatigue syndrome) was used with the aim of recruiting patients who would represent the type of patients presenting with chronic fatigue in general practice. Exclusion criteria were: (i) psychotic illness, (ii) organic brain syndrome or substance dependency, (iii) presence of physical health problems that could explain the fatigue or could contraindicate the use of graded exercise, and (iv) already in receipt of treatment for fatigue from a psychiatrist, counsellor, psychologist, community psychiatric nurse, physiotherapist or exercise specialist. Once the eligibility of the patients had been confirmed, a research associate gained written consent and collected the different measures to be used in the study.

### Clinical variables

The clinical condition of the patients was measured using the Chalder fatigue scale [10]. The Hospital Anxiety and Depression Rating Scale [11] was used to collect the depression and anxiety levels of the patients. The Work and Social Adjustment Scale (WASA), a self-rated measure, was used to evaluate the impact of the condition on the patient's life [12].

### Service use and costs

The use of services by patients in the six months before interview was measured using an adapted version of the Client Service Receipt Inventory [13]. Patients were asked to provide details of health and social care services used (including number of contacts and where appropriate the average duration). Services included primary and secondary healthcare contacts, complementary healthcare, social care, medication and tests/investigations. Additionally, patients were asked whether their contacts with professionals were due specifically to fatigue although all contacts were considered in the analysis. Unpaid informal care received by the patients as a consequence of their fatigue was also recorded. Patients stated how many hours per week family members or friends had helped with different tasks specifically because of the patient's fatigue.

These service use data were combined with appropriate unit costs for 2006/2007 obtained from national sources [14-16]. The unit costs of complementary and alternative therapies were obtained from other publications and sources [17-19]. Informal carers are not paid for their time, but clearly there is a value to their time. We used the replacement cost method for estimating the hidden cost of informal care, with the unit cost of a homecare worker being used as a proxy value (or 'shadow price'). This was based on the assumption that if the informal carer were unavailable a homecare worker would be the most likely formal service required to perform these tasks. The costs of different medicines were obtained from the British National Formulary for the year 2006 [20].

### Production costs

Patients were also asked about their salaries (if in work) and whether they had had to stop or reduce work due to their ill health and if so for how many days or hours per week during the previous six months. This information was used to calculate the productivity losses generated by the condition, assuming that individual levels of earnings reflect productivity, based on the human capital approach [21]. The result was multiplied by 0.8 to reflect the likelihood that reduced work time results in a less than proportionate reduction in productivity [22].

### Analyses

Costs were reported in the following three categories: (i) formal service costs, (ii) all service costs (i.e. including informal care), and (iii) total costs (i.e. including production costs). Service costs are also disaggregated into service categories (contacts with health and care professionals, inpatient care, tests and medication).

Multiple regression models were constructed to identify factors that explained variation in costs chronic fatigue. The dependent variables were the three measures of costs described above (formal services, all services and total costs). Demographic factors included in the models were gender, age, whether the patient cared for any dependants, and whether the patient lived alone. A variable reflecting the patients' perceptions of the cause of their fatigue (psychological or physiological) was included. Also included were the duration of the fatigue and functional impairment measured through the Work and Social Adjustment Scale (WASA),. Clinical factors included: total symptom score as measured by the Chalder Fatigue Scale, and depression and anxiety scores measured using the Hospital Anxiety and Depression Rating Scale. All variables were entered into the model in a single block. Cost data are often positively skewed, therefore generalised linear models with a log link and gamma errors were used [23].

### Sensitivity analyses

As there is uncertainty around a number of unit costs, sensitivity analyses were performed to examine how costs changed when the unit costs were varied. In the case of informal care, rather than the cost of a homecare worker used in the base-case analysis, it was assumed a cost per hour equivalent to the national minimum wage (£5.35). In the valuation of production costs it was assumed first that the fall in production was greater than the level of the wage, using a wage-production ratio of 1.2. Second, it was assumed that this ratio was 0.4, implying that 50% of employees would be able to make up for their lost time on their return to work.

Inclusion of the WASA in the regression models may mask the impact that other variables have on cost. Therefore, in sensitivity analyses we repeated the model but excluded the WASA scores.

### Ethics

The research protocol for this study was reviewed and approved by the Multi Centre Research Ethics Committee (MREC) West Midlands (MREC/02/7/71) and was approved by Lambeth, Southwark and Lewisham Primary Care Trusts (RDLSLG 142).

## Results

### Sample characteristics, service use and costs

The number of patients referred by GPs was 324. Of these, 100 did not meet the eligibility criteria and were excluded and 2 removed their consent. Therefore, data were collected for 222 patients. The characteristics of the sample are shown in Table 1. The majority were female, living with others and did not have any dependants. The mean duration of fatigue was high, at four-and-a-half years. However this was influenced in part by 8 participants who reported fatigue of over 20 years duration, being the median 26 months.

**Table 1:** Demographic and clinical characteristics of sample (n = 222).

Characteristic	N (%)
Male	48 (21.6)
Lives alone	41 (18.5)
Has dependants	73 (32.9)
Physical attribution of chronic fatigue	126 (56.8)
	
	**Mean (sd)**
Age (years)	39.8 (13.8)
Duration of fatigue (months)	52.9 (69.5)
Depression score^a ^(0-21)	7.8 (3.8)
Anxiety score^a ^(0-21)	9.2 (4.3)
Fatigue caseness^b ^(0-11)	9.1 (2.0)
Fatigue score^b ^(0-33)	24.6 (4.7)
Work and Social Adjustment Scale (0-32)	18.2 (7.8)

a; Hospital Anxiety and Depression (HAD) scale. b; Chalder Fatigue Questionnaire

All except one patient had contacts with their GP during the six-month period prior to interview (Table 2) - an expected finding given that population were recruited from primary care. There were a high number of contacts with psychologists and physiotherapists for other symptoms by those patients who accessed these services. Almost one-fifth of patients reported having used complementary healthcare services.

**Table 2:** Contacts with professionals and costs in six months prior to assessment (2006/07 £s).

Service	N	%	Mean number of contacts*	SD	Mean cost (all patients)	SD
General practitioner	221	99.5	4.9	2.9	231	139
Nurse	57	25.7	1.8	1.3	4	9
Pharmacist	22	9.9	2.4	1.6	8	29
Physiotherapist	17	7.7	4.2	3.5	5	24
Psychologist	17	7.7	9.4	7.2	45	205
Psychiatrist	8	3.6	2.5	1.8	13	79
Neurologist	8	3.6	1.1	0.4	6	34
Other doctor (including Accidents & Emergency)	63	28.4	2.3	1.7	64	137
Complementary and alternative therapy	40	18	6.5	7.2	43	143
Professionals allied to medicine	18	8.1	5	7.3	16	98

*by those using service

Table 3 shows the six-month mean service and lost production costs. Over half of the estimated cost of contacts with health and care professionals is related to non-chronic fatigue reasons. Apart from contacts with professionals, the other categories of healthcare costs account for a small proportion of cost. The cost of medication is very low at around £3 per month, with drugs for depression and anxiety accounting for most of this expenditure. Inpatient care, although expensive when it is used, accounted for just 5.9% of healthcare costs due to the relatively low numbers who were admitted. Formal service costs represented only 13% of the total.

**Table 3:** Mean cost of services and lost production in six months prior to assessment (2006/07 £s).

Category	N (%)	Mean (£)	SD	%
Healthcare	222 (100)	506	459	13.0
Contacts with professionals	222 (100)	436	385	86.3
Chronic fatigue related	212 (95.5)	194	259	44.5
Non-chronic fatigue related	191 (86.0)	243	306	55.6
Inpatient care	13 (5.9)	30	162	5.9
Tests/investigations	205 (92.3)	22	58	4.3
Medication	85 (38.3)	18	49	3.5

Informal care	67 (30.2)	1022	2459	26.4

Production costs	125 (56.3)	2350	3477	60.6

Total costs		3878	4573	

Informal care was required by almost a third of patients, and for these patients this amounted to an average of eight hours per week. Informal care accounted for just over a quarter (26.4%) of the total costs. More than half of the patients stated that their health had affected the number of hours that they were able to work or study and this resulted in a mean lost employment cost of £2350 during the six-month period. This accounts for just over 60% of the total economic cost.

### Factors associated with costs

**Figure 1:**
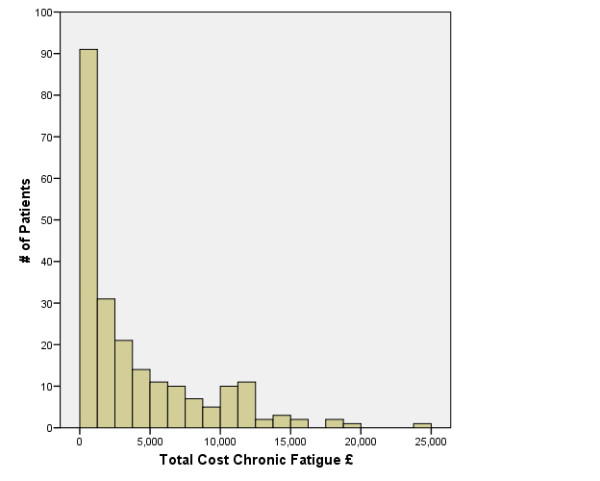
**Total Cost of Chronic Fatigue**.

The distribution of total costs is shown in Figure 1, illustrating the skewness of the data. Most of the sample had relatively low costs with a few patients having substantially higher costs. With regard to healthcare costs, having at least one dependant reduced health care cost by 23%, after controlling for the other characteristics, while higher levels of functional impairment resulted in higher costs (Table 4). When all service costs were included as the dependent variable, costs were higher for women than men, and higher costs were also associated with living with others and having a higher functional impairment score. Higher total costs (i.e. including production costs) were associated with greater severity of chronic fatigue and functional impairment. The variation explained by the models was 8%, 28% and 28% respectively.

**Table 4:** Generalised linear models to identify variables associated with cost variations.

	Health care costs (R^2 ^= 0.08)		Service costs (R^2 ^= 0.28)		Total Costs (R^2 ^= 0.28)	
**Variable**	**Coef**	**p**	**Coef**	**p**	**Coef**	**p**
Age (years)	0.001	0.763	-0.002	0.743	-0.004	0.551
Male (1 = yes, 0 = no)	-0.134	0.363	**-0.508**	**0.015**	-0.024	0.907
Dependants (1 = yes, 0 = no)	-0.235	0.083	0.297	0.121	0.078	0.674
Living alone (1 = yes, 0 = no)	-0.101	0.548	**-0.542**	**0.017**	0.015	0.945
Duration of fatigue (month)	-0.001	0.246	0.000	0.923	0.001	0.178
Physical attribution of fatigue (1 = yes, 0 = no)	-0.079	0.522	0.047	0.800	-0.078	0.668
Chronic fatigue score	0.008	0.598	0.038	0.111	**0.056**	**0.016**
Depression score	0.016	0.476	-0.024	0.453	0.031	0.300
Anxiety score	0.011	0.469	0.021	0.360	0.018	0.418
Functional impairment (WASA)	**0.022**	**0.022**	**0.062**	**<0.001**	**0.060**	**<0.001**

Service Costs are equal to Health care costs plus informal care costs. Total costs include service costs and production costs.

### Sensitivity analyses

If the national minimum wage is used to cost informal care rather than the cost of a homecare worker there is a 17.5% reduction in costs. There would be a 30% increase in costs if it was assumed that the fall in production was greater than the level indicated by wage rates and a 30% decrease if it was assumed that 50% of employees would be able to make up for their lost time on their return to work. The regression analysis without WASA scores (not shown) did not result in substantial differences in the health care cost model. In the analysis of service and total costs, the removal of the WASA variable from the model did modify the findings. The Chalder fatigue score became statistically significant for service costs and the variable indicating the level of depression was significant associated with total costs. The level of variation explained by the models was reduced significantly, with values of 6%, 12% and 15% respectively for health care, service and total costs.

## Discussion

The total economic cost associated with chronic fatigue is high, with informal care and lost productivity as a result of reduced work being the main contributors. The sensitivity analyses show that the total costs are extremely sensitive to the unit costs used for informal care and the method to value the lost production. Other studies have also shown the majority of costs due to chronic fatigue are 'hidden costs' such as informal care [9,24,25]. McCrone et al, reported informal care costs were more substantial than those due to lost productivity [24]. Other studies have highlighted the overall magnitude of the economic burden generated by chronic fatigue *syndrome*, estimated at £3.5 billion for the UK [26] and between $17 and $24 billion for the USA [27]. As far as we know this is the largest study of the costs of chronic fatigue conducted in the UK. It improves on previous work (e.g. [24]) in that a longer cost period is included and the distribution of costs is more appropriately reflected in the regression models.

Nearly a fifth of patients had used complementary and alternative therapies professionals, which is very similar to reports in the United States [28,29], Australia [30] and the UK [9,27]. This figure may be the result of patients who are turning to other avenues for care and help when faced with a health care system that does not satisfy their concerns and needs. This implies a group of fatigue patients feel the health service is not only denying people effective healthcare [3] but also means that they have to fund this care from their own pocket, increasing the financial burden on them and possibly exposing them to risk from unproven interventions practiced by (often) unregulated practitioners.

Over one-quarter of the variation in total service costs and costs including lost employment could be explained. This percentage is similar to the one found in a previous chronic fatigue study [24]. In the case of service costs, the fact that not living alone implies a higher cost might be explained by the effect of having a carer or helper available and its effect on informal care costs. Women had higher service costs than men, which may indicate a greater willingness for them to seek help for fatigue. Higher WASA scores implied higher costs of healthcare with and without informal care and also costs including lost production. This scale reflects the impact of fatigue on patients' lives and thus this association with costs is what would be expected. Together with the score that measures the severity of the fatigue these were the only two variables that could significantly explain the variation among individuals on total costs. Whilst associations between higher costs and greater severity and social problems were expected, it was of interest that other factors were not significant as the duration of fatigue or the age of the patients.

### Limitations

There are limitations to the study. First, there is a lack of a clear consensus as to how to calculate production costs. We used the most common method, the human capital approach, but different values for lost production were explored in sensitivity analyses. Questions in the CSRI were used to measure lost work time. The CSRI is adapted for each study but these questions have been used in numerous other studies. However, we did not measure the effect of reduced efficiency at work ('presenteeism') and so the production costs are likely to be an underestimate. Second, patient recall was used for measuring service utilisation and lost employment. This may not always be accurate, however data from patient recall can correlate well with data from other sources [31,32]. Third, the study recruited patients from 29 general practices in London and the South East of England. Although this ensured that urban, rural and inner city areas were included, the sample may still not be representative of the wider population, especially regarding salaries used to calculate the lost production, which tend to be higher in the London and the South East than other parts of the UK. There is also a small percentage of patients (12.6%) with a duration fatigue shorter than the 6 months period in which the use of services and lost productivity were collected. Nevertheless, when analysing the factors associated with cost variation, the duration of fatigue was included in the regression and therefore the results controlled by this factor. Finally, an economic evaluation comparing different therapeutic alternatives would be more useful for the purpose of choosing between them. Nevertheless, cost-of-illness studies, as this one, do indicate the general economic burden of illnesses and focus attention on those that result in high resource costs. This may act as an indicator for the prioritisation of economic research.

## Conclusions

The cost of formal services is only a small proportion of the overall costs for patients with chronic fatigue. People with chronic fatigue frequently receive care from their families and experience lost employment. These two features account for the greatest proportion of costs. These findings reinforce the point that healthcare professionals need to be aware of the real economic strain imposed on a person with chronic fatigue and their family, and that this can have serious implications such as the breakdown of relationships [7]. Documenting the economic burdens and how they are sustained can help health care workers to understand the causes and consequences, and show the empathy that is provided to patients with other conditions, but which is perceived to be lacking by patients with chronic fatigue syndrome [8].

## Competing interests

The authors declare that they have no competing interests.

## Authors' contributions

RSF did the data analysis, participated in the interpretation of the data and prepared the manuscript. PM contributed to the research proposal, reviewed the analysis, participated in the interpretation of the data and participated in the preparation of the manuscript. MH, MK and AND participated in the research proposal and the conduct of the study, and reviewed the manuscript. LR originated the idea for this study, developed the research proposal with MH and MK, led on the conduct of the study, participated in the interpretation of the data and participated in the preparation of the manuscript. All authors have read and approved the final manuscript.

## Pre-publication history

The pre-publication history for this paper can be accessed here:

http://www.biomedcentral.com/1472-6963/10/56/prepub
